# Iron overload and impaired iron handling contribute to the dystrophic pathology in models of Duchenne muscular dystrophy

**DOI:** 10.1002/jcsm.12950

**Published:** 2022-03-06

**Authors:** Francesca M. Alves, Kai Kysenius, Marissa K. Caldow, Justin P. Hardee, Jin D. Chung, Jennifer Trieu, Dominic J. Hare, Peter J. Crouch, Scott Ayton, Ashley I. Bush, Gordon S. Lynch, René Koopman

**Affiliations:** ^1^ Centre for Muscle Research, Department of Anatomy and Physiology The University of Melbourne Melbourne Victoria Australia; ^2^ Department of Biochemistry and Pharmacology The University of Melbourne Melbourne Victoria Australia; ^3^ Melbourne Dementia Research Centre, The Florey Institute of Neuroscience and Mental Health The University of Melbourne Melbourne Victoria Australia; ^4^ Monash eResearch Centre Monash University Clayton Victoria Australia

**Keywords:** Muscular dystrophy, Muscle metabolism, Iron, Oxidative stress

## Abstract

**Background:**

Oxidative stress is implicated in the pathophysiology of Duchenne muscular dystrophy (DMD, caused by mutations in the dystrophin gene), which is the most common and severe of the muscular dystrophies. To our knowledge, the distribution of iron, an important modulator of oxidative stress, has not been assessed in DMD. We tested the hypotheses that iron accumulation occurs in mouse models of DMD and that modulation of iron through the diet or chelation could modify disease severity.

**Methods:**

We assessed iron distribution and total elemental iron using LA‐ICP‐MS on skeletal muscle cross‐sections of 8‐week‐old Bl10 control mice and dystrophic *mdx* mice (with moderate dystrophy) and dystrophin/utrophin‐null mice (*dko*, with severe dystrophy). In addition, *mdx* mice (4 weeks) were treated with either an iron chelator (deferiprone 150 mg/kg/day) or iron‐enriched feed (containing 1% added iron as carbonyl iron). Immunoblotting was used to determine the abundance of iron‐ and mitochondria‐related proteins. (Immuno)histochemical and mRNA assessments of fibrosis and inflammation were also performed.

**Results:**

We observed a significant increase in total elemental iron in hindlimb muscles of *dko* mice (+50%, *P* < 0.05) and in the diaphragm of *mdx* mice (+80%, *P* < 0.05), with both tissues exhibiting severe pathology. Iron dyshomeostasis was further evidenced by an increase in the storage protein ferritin (*dko*: +39%, *P* < 0.05) and ferroportin compared with Bl10 control mice (*mdx*: +152% and *dko*: +175%, *P* < 0.05). Despite having features of iron overload, dystrophic muscles had lower protein expression of ALAS‐1, the rate‐limiting enzyme for haem synthesis (*dko* −44%, *P* < 0.05), and the haem‐containing protein myoglobin (*dko* −54%, *P* < 0.05). Deferiprone treatment tended to decrease muscle iron levels in *mdx* mice (−30%, *P* < 0.1), which was associated with lower oxidative stress and fibrosis, but suppressed haem‐containing proteins and mitochondrial content. Increasing iron via dietary intervention elevated total muscle iron (+25%, *P* < 0.05) but did not aggravate the pathology.

**Conclusions:**

Muscles from dystrophic mice have increased iron levels and dysregulated iron‐related proteins that are associated with dystrophic pathology. Muscle iron levels were manipulated by iron chelation and iron enriched feed. Iron chelation reduced fibrosis and reactive oxygen species (ROS) but also suppressed haem‐containing proteins and mitochondrial activity. Conversely, iron supplementation increased ferritin and haem‐containing proteins but did not alter ROS, fibrosis, or mitochondrial activity. Further studies are required to investigate the contribution of impaired ferritin breakdown in the dysregulation of iron homeostasis in DMD.

## Introduction

Duchenne muscular dystrophy (DMD) is the most common and severe of the muscular dystrophies.^1^ It is an X‐linked recessive disease caused by mutations in the *dystrophin* gene (DMD; locus Xp21.2), resulting in the absence of the dystrophin protein, rendering muscle fibres fragile and prone to injury. Without dystrophin to stabilize the membrane and connect the contractile filaments to the basal lamina, muscle contractions can induce micro‐lesions in the membrane that impair Ca^2+^ homeostasis and lead to aberrant reactive oxygen species (ROS) production and inflammation.^1^ While not the initial cause of the disease, oxidative stress correlates with the severity of the dystrophic pathology and is a common therapeutic target for the muscular dystrophies.^2^

Enhanced ROS generation leading to lipid peroxidation has been suggested as a mechanism of muscle degeneration in muscular dystrophies.^3^ Studies have reported Ca^2+^ accumulation in muscle biopsies of DMD patients^4^ and investigated the role of elevated intracellular Ca^2+^ ([Ca^2+^]_i_) in the progression of the dystrophic pathology and its role in the production of ROS.^5^ However, elevated [Ca^2+^]_i_ is not the only factor (or element) associated with ROS production and the progression of the dystrophic pathology. Our recent study demonstrated a link between dysregulated iron homeostasis with muscle atrophy and mitochondrial dysfunction in sarcopenia,^6^ but to date, no studies have assessed whether iron, an abundant pro‐oxidant, is similarly elevated in dystrophic skeletal muscle.

In 1984, Clark proposed treating DMD with the iron chelator deferoxamine to reduce free‐iron‐induced generation of ROS and restore the redox balance with the membrane lipid antioxidant tocopherol (vitamin E).^7^ This hypothesis was based on observations of vitamin E deficiency,^8^ oxidative stress in human erythrocytes,^9^ and increased lipid peroxidation products (malonyldialdehyde; MDA), glutathione peroxidase and glutathione reductase in dystrophic skeletal muscles.^10^ Three decades later, the effect of iron chelator (deferoxamine) treatment was assessed in *mdx* mice, the most commonly used model for studying DMD.^11^ Deferoxamine significantly decreased oxidative damage as evident from reduced levels of 4‐hydroxynonenal (4HNE, another lipid peroxidation product) and dihydroethidium (DHE) in the severely affected diaphragm muscle of *mdx* mice. Reduced oxidative stress and inflammation (decreased NF‐κB) in deferoxamine‐treated mice were associated with less muscle damage and preserved muscle strength (grip strength).^11^ That study did not measure iron or iron‐related proteins to explore the role of iron dyshomeostasis.

It is well established that free iron promotes generation of ROS. Both Fe^2+^ and Fe^3+^ undergo redox reactions that can result in the generation of hydroxyl radicals, which are dangerous in biological systems as they can adduct a range of biomolecules: carbohydrates, nucleic acids, lipids and amino acids.^12^ We recently utilized laser ablation‐inductively coupled‐mass spectrometry (LA‐ICP‐MS), a comprehensive method for imaging the spatial distribution of metals in tissue cross sections,^13^ to visualize and quantify the iron content in muscles of aged mice.^6^ LA‐ICP‐MS combines a focused ultraviolet laser beam with mass spectrometry‐based methods, which are the most sensitive techniques capable of measuring biologically relevant metals.^14^ This innovative technique can be applied to any tissue to assess changes in metal homeostasis within fine anatomical structures. To our knowledge, we are the first to use this technique to assess changes in iron homeostasis in muscle tissue from dystrophic mice. In other models of muscle atrophy, such as in ageing and cancer cachexia (gastric cancer), iron overload (iron and ferritin elevation) has been associated with markers of oxidative stress,^6^, ^15^ contributing to mitochondrial dysfunction and muscle atrophy (sarcopenia).

To test the hypothesis that iron burden contributes to oxidative stress and is associated with the severity of the dystrophic pathology, we used *mdx* mice, a genetic homologue of the human disease and the most widely used model of DMD. Compensatory upregulation of utrophin mitigates pathology in the limb muscles of *mdx* mice; however, the diaphragm muscle of *mdx* mice lacks this compensation and has a severe pathology. We also utilized *dko* mice which lack both dystrophin and utrophin, thus exhibiting a more severe muscle pathology in hindlimbs than *mdx* mice.^16^ For a progressive phenotype we analysed the hindlimb muscles of *mdx* and *dko* mice. Subsequent interventions were performed in the *mdx* mice; however, analysis was focused on the diaphragm due to its severity and thus closer representation to the DMD pathology. We sought to (i) characterize iron homeostasis in DMD using LA‐ICP‐MS; and (ii) modulate iron levels either through the diet or chelation to determine whether iron levels affect the disease severity.

## Methods

### Animals

All experiments were approved by the Animal Ethics Committee of The University of Melbourne and conducted in accordance with the Australian code of practice for the care and use of animals for scientific purposes as stipulated by the National Health and Medical Research Council (NHMRC, Australia). Male *mdx* mice (4–5 weeks old) were sourced from the Animal Resources Centre (ARC), Canning Vale, WA, Australia. During the intervention, all mice were housed under a 12:12 h light–dark cycle with temperature control in the Biological Research Facility (The University of Melbourne) and monitored weekly. Male *dko* mice were bred as described previously.^17^ Briefly, female *mdx* breeders (sourced from the ARC) were bred with male utrophin knockout mice (Utrn^−/−^). F1 generation females were then mated with Utrn^−/−^ males, with an expected male dystrophin/utrophin‐null (*dko*) yield of 25%.

### Experimental outline

#### Deferiprone administration

Deferiprone (DFP; 3‐hydroxy‐1, 2‐dimethyl‐4(1*H*)‐pyridine; Sigma‐Aldrich, NSW, Australia), is a lipid‐soluble iron chelator used for treatment of iron overload in thalassaemia.^18^ Deferiprone binds to iron and can remove excess iron from the body. Mice were given free access to DFP dissolved in the drinking water (1 mg/mL). On average mice drank 3 mL per day. Male *mdx* mice (4–5 weeks old) were treated with 150 mg/kg DFP^6^ for 4 weeks.

#### Iron administration

Male *mdx* mice (4 weeks old) received an iron‐enriched feed (modified SF07‐082 Semi‐Pure Rodent Diet) ad libitum containing 1% added iron as carbonyl iron (Specialty Feeds, WA; Australia),^19^ or a control diet (AIN93G; Specialty Feeds, WA; Australia) for 4 weeks.

### Whole body functional assessments

#### Grip strength

Forelimb strength was assessed using a grip strength meter (Columbus Instruments, Columbus, Ohio, USA).^6^ Briefly, mice grasped a triangular metal ring connected to a force transducer and the tail was pulled gently until the grip was broken. Peak force was measured in kilograms (kg). Each mouse performed the test five times within 2 min with adequate rest time (30 s) between attempts. The maximum force achieved was normalized to their body mass at the time of assessment.

#### Body composition

At the end of treatment, whole body composition was analysed using magnetic resonance relaxation analysis of live body composition of fat tissue, lean tissue, free water, and total water (LF50, Bruker, USA).

#### Glucose tolerance test

Mice underwent a glucose tolerance test following day 23 of treatment of DFP. Mice were fasted overnight (~16 hr) prior to a glucose challenge (1 g/kg body mass of 0.1 g/mL glucose (Sigma‐Aldrich) dissolved in sterilized saline, via an intraperitoneal [i.p.] injection). Baseline blood sampling was conducted with a glucose meter (ACCU‐CHEK Performa). Following the i.p. injection of glucose, mice were returned to their usual housing cages. Blood glucose readings were measured again 15, 30, 60, 90, and 120 min following the i.p. injection by re‐puncturing the tail vein to acquire a droplet of blood.

### Endpoint measurements

#### Tissue collection

At the end of the treatment period, mice were anaesthetised with 0.2 mL of sodium pentobarbitone (Nembutal; 60 mg/kg; Sigma‐Aldrich, i.p. injection) and killed via cervical dislocation followed by cardiac excision. The epididymal fat pad, heart, diaphragm and skeletal muscles of the hindlimbs; tibialis anterior (TA), gastrocnemius (GAST), extensor digitorum longus (EDL), quadriceps (QUAD), plantaris (PLAN), and soleus (SOL) were excised, weighed, snap frozen in liquid nitrogen and stored at −80°C for later analysis. Half of the TA and diaphragm muscles were mounted in optimal cutting temperature embedding compound (Tissue‐Tek, Sakura Finetek, Canada), frozen in liquid‐nitrogen‐cooled isopentane, and stored at −80°C for later analysis. The tibia bone was dissected, and length measured using digital callipers.

#### Blood serum collection

Blood was collected from the intact heart. Whole blood droplets were used to measure resting blood glucose using a blood glucose meter (Accu‐Chek, Australia) and resting haematocrit levels (HemoCue® Hb 201+ System, Australia).

### Biochemical analysis

#### Protein extraction and immunoblotting

**Figure 1:**
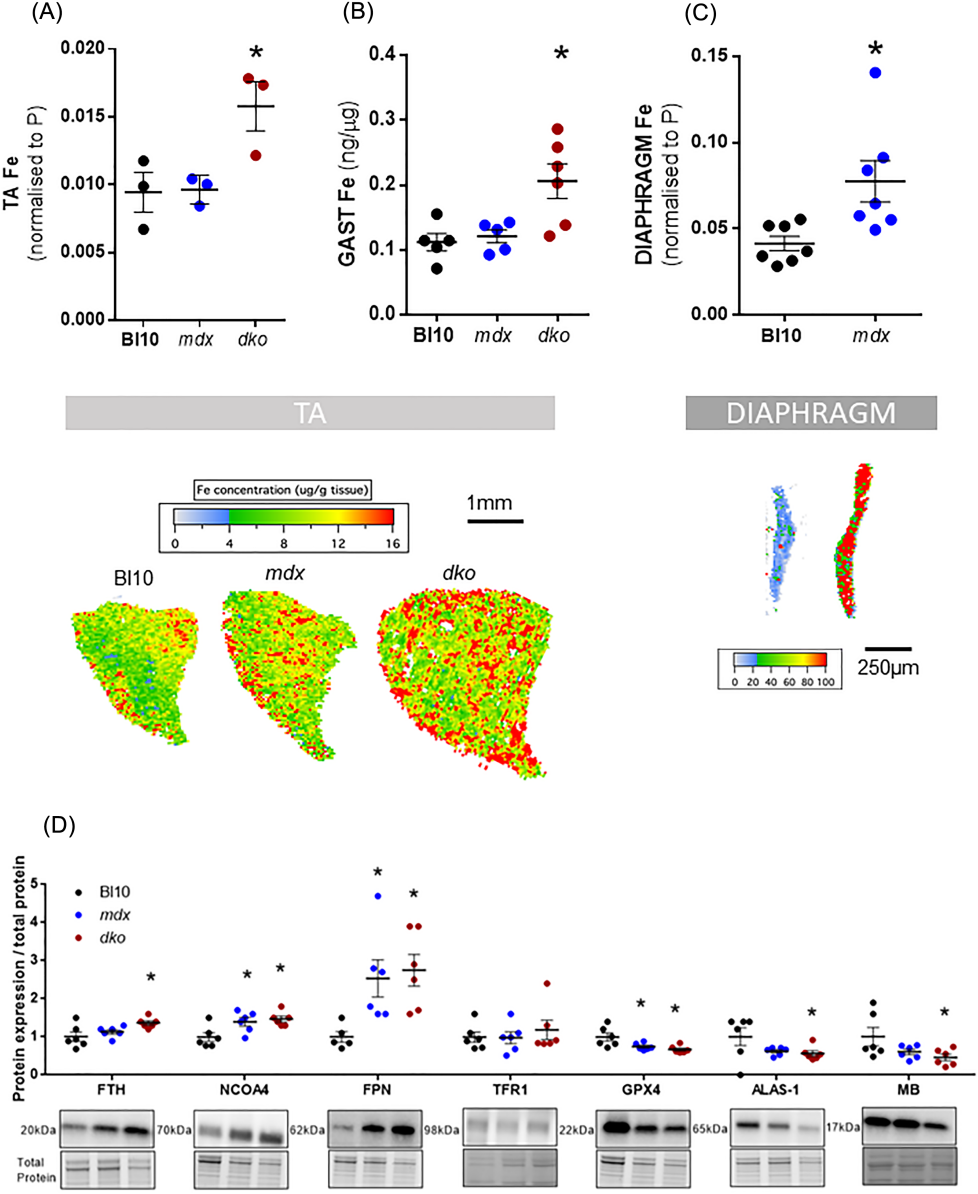
Severely dystrophic muscles from *dko* (dystrophin‐utrophin knockout) and *mdx* mice show iron (Fe) dysregulation. Total elemental iron (normalized to phosphorus [P]), assessed via LA‐ICP‐MS, was elevated in tibialis anterior (TA) muscles in *dko* mice (*n* = 3) (*A*, with representative LA‐ICP‐MS images below the graph) and gastrocnemius muscles (*n* = 6) (*B*). The diaphragm in *mdx* mice had significant elevations of total iron (*C*, with representative LA‐ICP‐MS images below the graph). Panels (*A*) and (*C*) are quantified from LA‐ICP‐MS muscle cross‐sections (normalized to P) and (*B*) is microdroplet analysis of muscle homogenates normalized to protein concentration. Immunoblotting of gastrocnemius (GAST) muscle showed increased FTH, NCOA4, FPN and decreased GPX4, MB and ALAS‐1 and no change in TFR1 in muscles of *dko* mice (*D*). Data presented as mean ± SEM. Data were analysed using one‐way ANOVAs with Tukey's (*A*) post hoc test. *P ≤ 0.05.

TA muscles (*Figure* 1) and diaphragm muscles (*Figures* 3 and 5) (10–30 mg) were homogenized in ice‐cold buffer (Tris buffered saline, TBS; 50 mM Tris‐Cl, pH 7.6; 150 mM NaCl; Sigma‐Aldrich; in Milli‐Q H_2_O) containing 2% (v/v) Complete™ Ethylenediaminetetraacetic acid (EDTA)‐free protease inhibitor (Roche, Hawthorn, Australia) in tubes containing a 1.0 mm zirconia/silica beads (Daintree Scientific, Tasmania, Australia) as described previously.^6^ Total protein concentration of the supernatant was determined using the Bio‐Rad DC protein assay kit per the manufacturer's instructions (Bio‐Rad Laboratories, NSW, Australia).

Homogenates (0.5–1 g/L) were denatured at 95°C in 4 × Laemmli sample buffer containing dithiothreitol (DTT; Sigma‐Aldrich) for 5 min. Protein (5–10 μg) was separated by 4–20% SDS‐PAGE (Criterion TGX Stain‐Free Precast Gels; Bio‐Rad Laboratories) for 1.5 h at 150 V. Proteins were transferred onto 0.2 μM nitrocellulose membranes using the Transblot Turbo system (Bio‐Rad Laboratories), with a constant output of 25 V for 7 min. Proteins were visualized prior to and following transfer using the ChemiDoc™ MP Imaging System (Bio‐Rad Laboratories. Membranes were blocked using 5% bovine serum albumin (BSA) in tris buffered saline with 1% Tween 20 (TBST) at room temperature (RT) for 2 h then incubated in primary antibodies in 5% BSA in TBST overnight at 4°C [ferroportin, ALAS‐1, cytochrome *c*, ferritin, GPX4, myoglobin, PGC1‐α (Abcam, United Kingdom), VDAC (Invitrogen, United States), NCOA4 (Santa Cruz Biotechnology, United States), and TFR1 (Alpha Diagnositc, United States)]. Membranes were washed five times with TBST (5 min each) prior to incubation with an appropriate horseradish peroxidase (HRP) conjugated secondary antibody. Membranes were washed five times with TBST and proteins were visualized with SuperSignal® West Femto Maximum Sensitivity Substrate (Thermo Fisher Scientific Inc., VIC, Australia) using the ChemiDoc™ MP Imaging system (Bio‐Rad Laboratories). The density of bands was quantified using Image Lab software (Bio‐Rad Laboratories) and normalized to total protein. Images of stain free gels were used to quantify total protein loading for each lane.

#### Citrate synthase enzyme activity

Diaphragm muscles were homogenized using a cooled Precellys24® tissue homogeniser (2 cycles of 15 s at 5500 rpm; Sapphire Bioscience, NSW, Australia) in homogenizing buffer (10 mM Tris HCl (pH 7.4), 100 mM NaCl, 1 mM EGTA, 1 mM EDTA, 1% Triton X, 10% glycerol, 0.1% SDS, 20 mM Na_4_P_2_O_7_, 2 mM Na_3_VO_4_, 1 mM NaF, 0.5% sodium deoxycholate, and 1 mM PMSF). The homogenates were frozen under liquid nitrogen and thawed four times to disrupt the mitochondria to expose CS. Total muscle protein was determined in triplicate by the Bio‐Rad DC protein assay kit per the manufacturer's instructions (Bio‐Rad Laboratories, NSW, Australia), and the protein concentration of all samples equalized. Citrate synthase activity was determined, normalized to total protein content, and expressed in nanomoles per milligram protein per minute as described previously.^6^

#### Ribonucleic acid extraction and quantitative polymerase chain reaction analyses

Total RNA was extracted from 10–20 mg of diaphragm muscle using a commercially available kit, according to the manufacturer's instructions (RNeasy Mini Kit, QIAGEN, VIC, Australia). RNA quality and concentration were determined using the Nanodrop 1000 (Thermo‐Fisher Scientific, VIC, Australia). First‐strand cDNA was generated using the iScript™ as previously described.^6^ Primer sequences are listed in *Table* 1 or described elsewhere.^6^

**Table 1:** Primer sequences were designed using NCBI primer‐BLAST using sequences accessed through GenBank and checked for specificity using nucleotide BLAST search

Gene name	GenBank accession no.	Forward sequence (5′‐3′)	Reverse sequence (5′‐3′)	Amplicon length
*Mmp9*	NM_013599.5	ATGTCTCGCGGCAAGTCTTC	CCGACTTTTGTGGTCTTCCCC	332
*Vegf*	NM_001025250	CAGGCTGCTGTAACGATGAA	GCATTCACATCTGCTGTGCT	140
*Timp1*	NM_011593.2	CATCCTCTTGTTGCTATCAC	CATGAATTTAGCCCTTATGACC	112
*Timp3*	NM_011595.2	GCTAGAAGTCAACAAATACCAG	TAGTAGCAGGACTTGATCTTG	174

*Mmp9*, matrix metalloproteinase‐9; *Vegf*, vascular endothelial growth factor; *Timp1*, tissue inhibitor of metalloproteinases 1; *Timp3*, tissue inhibitor of metalloproteinases 3.

#### Histology

**Figure 2:**
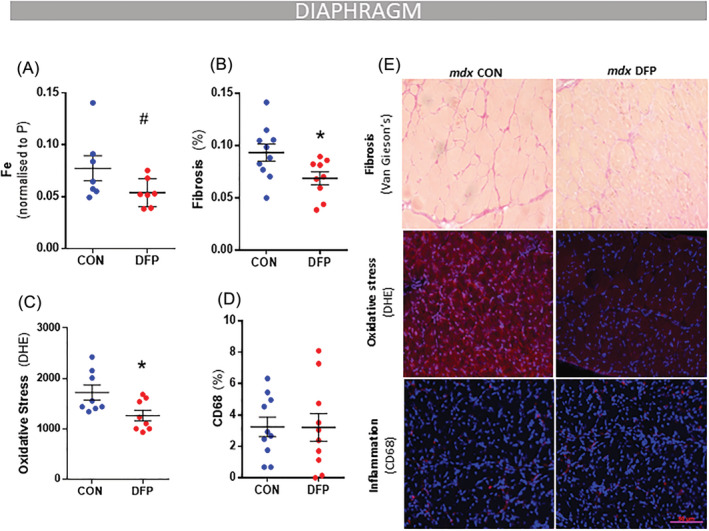
Iron chelation treatment with DFP treatment attenuates aspects of dystrophic pathology. *mdx* mice (4 weeks) were given access to drinking water with or without deferiprone (DFP: 150 mg/kg/day) for 4 weeks. DFP treatment reduced iron levels (analysed via LA‐ICP‐MS) in the diaphragm muscle (*A*). Iron chelation also reduced fibrosis (Van Gieson's) (*B*) and oxidative stress (dihydroethidium; DHE, *C*) but had no significant impact on inflammation (CD68% nuclei, *D*) in the diaphragm muscle. Representative images of stains are shown in panel (*E*). Data presented as mean ± SEM. Data were analysed using Student's *t*‐test. *P ≤ 0.05 and #*P* ≤ 0.1. *N* = 8–10.

Serial sections (10 and 30 μm) were cut transversely through the TA muscles (*Figure* 1) and diaphragm muscles (*Figures* 2 and 4) (10–30 mg) using a refrigerated (−20°C) cryostat (CTI Cryostat; IEC, Needham Heights, MA, USA). Sections (10 μm) were stained with haematoxylin and eosin (H&E) to determine general muscle architecture^17^ and CD68 for determination of macrophage infiltration.^20^ For Pax7/Ki67+ (muscle stem cell characterization), sections were fixed in 4% paraformaldehyde (PFA) for 10 min at RT, washed twice in PBST (0.1% Triton X) for 5 min, and then boiled in citrate buffer pH 7.4 for 10 min in a pressure cooker. Sections were blocked in 3% BSA/PBST for 2 h at RT, then incubated overnight in primary antibody in blocking solution at 4°C. Secondary antibodies were added for 1.5 h at RT, mounted and then imaged on a Zeiss LSM880 microscope (AxioImager Z2; Carl Zeiss, Wrek Göttingen, Germany) controlled by Zen 2.3 software. Digitally captured images (×10 magnification) with a minimum of three fields‐of‐view per muscle cross‐section were processed and analysed.

Digital images of CD68/DAPI and Van Gieson's histological stain in *Figure* 4 and CD68/DAPI in *Figure* 2 were obtained using an upright microscope with camera (Axio Imager D1; Carl Zeiss), controlled by AxioVision AC software (AxioVision AC Rel. 4.8.2; Carl Zeiss Imaging Solutions). Images were quantified with AxioVision 4.8.2 software. Digitally captured images (×120 magnification) with a minimum of three fields‐of‐view per muscle cross‐section were processed and analysed.

For all other imaging, slides were submitted for digitalisation at the Australian Phenomics Network Histopathology and Organ Pathology slide scanning service (Melbourne, Australia; Manufacturer: 3D Histec, Model: Pannoramic SCAN II. Objective: Carl Zeiss Plan‐Apochromat 20x/NA 0.8, using a Point Grey Grasshopper 3CCD monochrome camera with LED‐based RGB illumination unit). Scans were viewed and images taken using Caseviewer Software. Subsequent analysis was performed using FIJI Software.^21^

#### Laser ablation‐inductively coupled plasma‐mass spectrometry

Laser ablation‐inductively coupled plasma‐mass spectrometry experiments were carried out as described previously for tissue imaging on 30 μm TA and diaphragm tissue sections^22^ and GAST μ‐droplet analysis.^23^ A NewWave Research NWR213 laser ablation system (Kennelec Scientific, Mitcham, Australia) with a standard two‐volume cell was used for all analyses. Argon was used as the carrier gas (1.2 L/min). All measurements were performed using an Agilent 8800 triple quadrupole ICP‐(QQQ)‐MS system with ‘cs’ lenses. The ICP‐QQQ‐MS system used was previously optimized to ‘no gas’ tuning parameters to maximize ion focusing and transmission via the Q2 ion‐guide and collision/reaction cell.^24^

For tissue imaging, silica glass microscope slides (Menzel‐Gläser Superfrost® Plus; Thermo‐Fisher Scientific, Scoresby, Australia) with 30 μm thick muscle sections were placed in a 10 × 10 cm ablation cell together with matrix‐matched elemental standards for quantitative analysis. Muscle sections were ablated with a 60 μm square beam laser using a series of rasters and a scanning speed of 240 μm/s and elemental data collected for carbon (^13^C), phosphorus (^31^P) and iron (^56^Fe). The measured iron in each pixel was normalized to the corresponding high intensity (>8%) phosphorus signal to minimize inter‐run variability when using LA‐ICP‐MS. For high resolution images for Perls' stain matching, selected sections were ablated with a 15 μm square beam laser using a series of rasters and a scanning speed of 30 μm/s.

For μ‐droplet analysis,^22^ up to 150 droplets (0.5 μL) of samples (normalized to protein concentration) and standards were deposited onto microscope slides manually in triplicate. Standards and samples were deposited in rows within the 5 × 2.5 cm working area of a single slide and then air‐dried in a particle‐free environment overnight. A square beam 100 μm wide scanned at 200 μm/s and 0.3–0.5 J/cm fluence laser power was used to remove all deposited material while not ablating the supporting glass, resulting in a total analysis time of approximately 8–14 h per slide. The ICP‐QQQ‐MS system was configured to measure the mass‐to‐charge (m/z) ratios for elements carbon (^13^C), phosphorus (^31^P), magnesium (^24^Mg), calcium (^44^Ca), and iron (^56^Fe).

Single line scans from LA‐ICP‐MS analysis (as .csv files) were collated into hyperspectral images using iolite (v.3; The University of Melbourne, Parkville, Australia) with the Biolite add‐on for image analysis (*Figure* 1E; see Hare *et al*.^22^ for a visual tutorial). Modifications to the image analysis code were made using Igor Pro (v7; WaveMetrics, Inc., Portland, USA). Region of interest (ROI) tool was used to extract mean (x¯) counts per second (CPS) for each m/z from the desired area. For μ‐droplet analysis, external calibration was performed via linear regression analysis using a 4‐point (including matrix blank) calibration curve in Prism (v7, GraphPad, La Jolla, USA). All μ‐droplet dilution series were analysed in triplicate, or as otherwise stated.

#### Inductively coupled plasma‐mass spectrometry

Briefly, homogenate samples were lyophilised and then digested with nitric acid (65% Suprapur, Merck, St. Louis, MO, USA) overnight, followed by heating at 90°C for 20 min using a heat block. Samples were then removed from the heat block and an equivalent volume of hydrogen peroxide (30% Aristar, BDH, Radnor, PA, USA) added to each sample. Once samples had finished digesting, they were heated for a further 15 min at 70°C. Samples were then diluted with 1% nitric acid diluent. Measurements were made using an Agilent 7700 series ICP‐MS instrument under routine multi‐element operating conditions using a helium reaction gas cell. The instrument was calibrated using 0, 5, 10, 50, 100, and 500 ppb of certified multi‐element ICP‐MS standard calibration solutions (ICP‐MS‐CAL2‐1, ICP‐MS‐CAL‐3, and ICP‐MS‐CAL‐4, Accustandard, New Haven, CT, USA) for a range of elements, and we also utilized a certified internal standard solution containing 200 ppb of Yttrium (Y89) as a control (ICP‐MS‐IS‐MIX1‐1, Accustandard).

### Statistical analysis

Data were analysed with GraphPad Prism software version 7 (GraphPad Software Inc., La Jolla, CA). Unpaired t‐tests were used for comparisons between two groups. For comparisons between more than two groups, a one‐ or two‐way analysis of variance (ANOVA) was used, as appropriate, with Tukey's post hoc multiple comparison test when significance was detected. The level of significance was set at *P* < 0.05 for all comparisons. All values are presented as means ± SEM.

## Results

### Iron is increased in dystrophic muscles

Our data showed significantly higher iron abundance in the TA muscles of severely dystrophic *dko* mice (+50%, *P* < 0.05; *Figure* 1A). Similarly, iron levels were significantly higher in lysates from gastrocnemius muscles of dystrophic mice (*dko*: +50%, *P* < 0.05; *Figure* 1B). In *mdx* mice, limb muscles have a comparatively mild pathology, and these muscles did not display increases in iron. However, in the diaphragm muscle, which is severely affected in *mdx* mice, there was a significant increase of total iron (+80%, *P* < 0.05; *Figure* 1C).

The increase in iron was associated with changes in proteins involved in iron homeostasis. The storage protein ferritin (FTH) was increased in dystrophic muscle (*dko*: +39%, *P* < 0.05; *Figure* 1D) along with nuclear receptor coactivator 4 (NCOA4), a protein that regulates ferritin breakdown via autophagy (*mdx*: +40% and *dko*: +48%, *P* < 0.05; *Figure* 1D), and ferroportin (FPN), which mediates iron export (*mdx*: +152% and *dko*: +175%, *P* < 0.05; *Figure* 1D). Iron import protein transferrin receptor 1 (TFR1) was not altered in dystrophic muscles. Despite having features of iron overload, dystrophic muscles had lower protein expression of the rate‐limiting enzyme for haem synthesis, (*dko* −44%, *P* < 0.05; *Figure* 1D) and the haem‐containing protein myoglobin (MB; *dko* −54%, *P* < 0.05; *Figure* 1D). In addition, glutathione peroxidase 4 (GPX4), an enzyme that suppresses membrane phospholipid peroxidation, was also significantly decreased in dystrophic muscle (*mdx*: −26% and *dko* −33%, *P* < 0.05; *Figure* 1D).

### Deferiprone treatment attenuates aspects of the dystrophic pathology in mdx mice but impairs mitochondrial function

**Figure 3:**
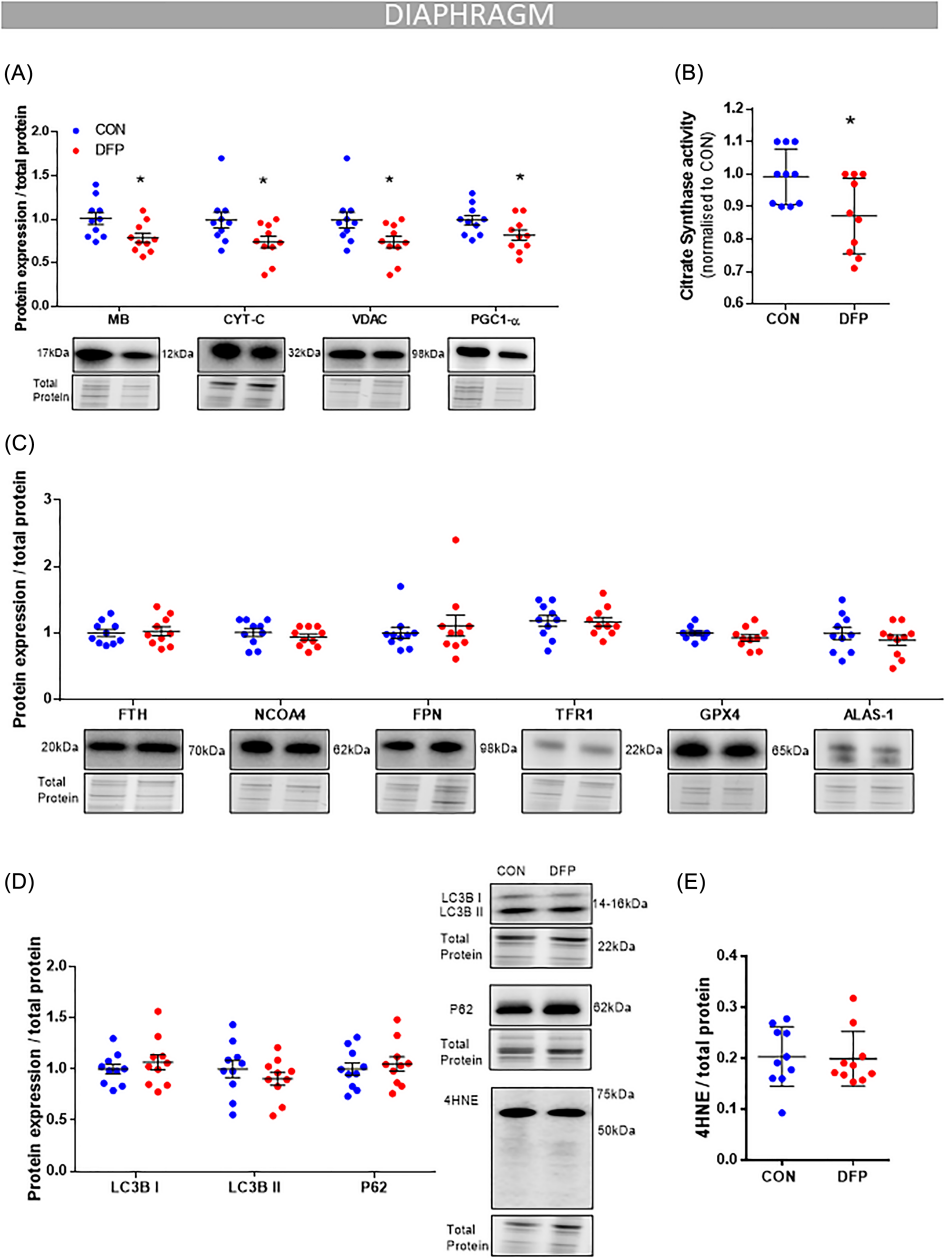
DFP treatment reduces mitochondrial and haem‐containing proteins. *mdx* mice (4 weeks) were given access to drinking water with or without deferiprone (DFP: 150 mg/kg/day; *n* = 10) for 4 weeks. Immunoblotting analysis in the diaphragm muscle showed that DFP reduced two proteins that contain haem units, MB and CYT‐C, along with mitochondrial markers VDAC and PGC1‐α (*A*). Citrate synthase activity was also reduced (*B*). There was no change in FTH, NCOA4, FPN, TFR1, GPX4, and ALAS‐1 (*C*), LC3BI, LC3BII, P62 (*D*), or 4HNE (*E*). Data presented as mean ± SEM. Data were analysed using Student's *t*‐test. *P ≤ 0.05. *N* = 10 in each group.

We tested the hypothesis that iron chelation would ameliorate oxidative stress and fibrosis in *mdx* mice. DFP treatment did not affect growth (Supporting Information, *Figure* S1A), body composition (*Figure* S1B), individual muscle masses (*Figure* S1C), maximum grip strength (*Figure* S1D), or blood glucose (*Figure* S1E). DFP lowered total iron in the diaphragm muscle without reaching statistical significance (−30%, *P* < 0.1; *Figure* 2A) and did not affect muscle ferritin levels (*Figure* 3C). DFP reduced superoxide levels (DHE: −27%, *P* < 0.05; *Figure* 2C) and fibrosis (−26%, *P* < 0.05; *Figure* 2B) but did not affect macrophage infiltration (CD68 positive cells) (*Figure* 2D) or Pax7/Ki67+ (*Figure* 2) in muscle cross‐sections (*Figure* 2D). Tumour necrosis factor α (*TNFα*) mRNA was suppressed (−36%, *P* < 0.05; *Table* S1) and *F480* mRNA increased (+184%, *P* < 0.05; *Table* S1) in diaphragm muscles of DFP‐treated mice. No significant changes were seen in other inflammatory or fibrosis mRNA markers (*Ccl2*, *Socs3*, *Il6*, *Cd80*, *Col1a1*, *Col2a1*, *Col3a1*, *Mmp2*, *Mmp9*, *Tgfb1*, *Tgfb2*, *Tgfb3*, *Vegf*, *Timp1*, and *Timp3*).

Deferiprone treatment suppressed haem‐containing proteins, reducing the protein abundance of both myoglobin (MB; −21%, *P* < 0.05; *Figure* 3A) and cytochrome *c* (CYT‐C; −26%, *P* < 0.05; *Figure* 3A). These observations coupled with suppressed superoxide levels (*Figure* 2C) were consistent with impaired mitochondrial activity, which was confirmed by reduced citrate synthase enzyme activity (−13%, *P* < 0.05; *Figure* 3B) in diaphragm muscles of DFP treated mice as well as reduced protein expression of key mitochondrial markers VDAC (−26%, *P* < 0.05; *Figure* 3A) and PGC1‐α (−19%, *P* < 0.05; *Figure* 3A). No significant changes were seen in autophagy markers microtubule‐associated protein 1A/1B‐light chain 3 (LC3BI/II), sequestosome‐1 (P62) (*Figure* 3E) or marker of lipid peroxidation 4‐hydroxy‐2‐nonenal (4HNE, *Figure* 3E).

### Increasing dietary iron does not affect dystrophic pathology but increases the expression of ferritin and haem‐containing proteins

**Figure 4:**
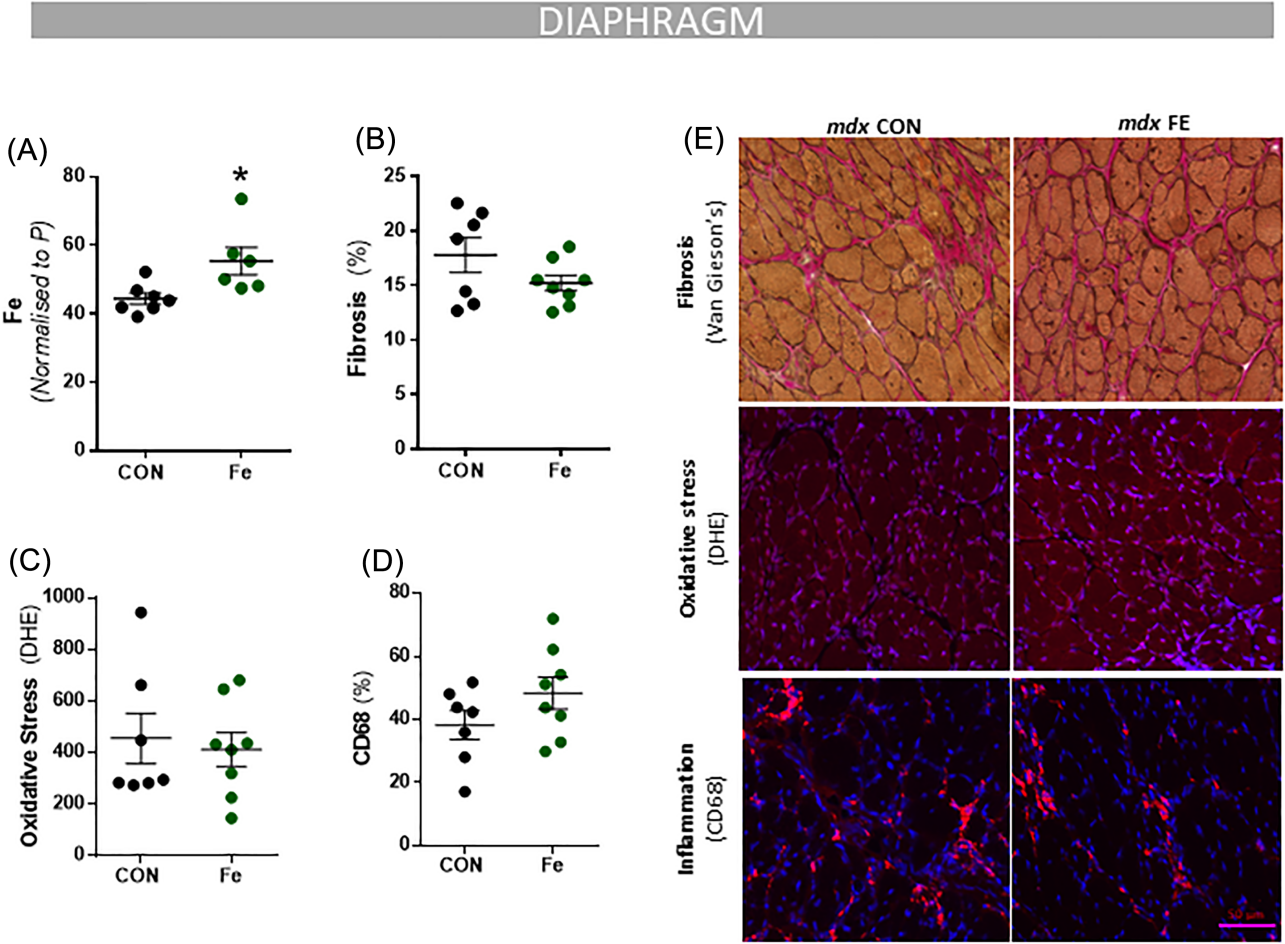
Fe supplementation does not affect dystrophic pathology in *mdx* mice. *mdx* mice (4 weeks) were fed an iron enriched feed containing 1% added Fe as carbonyl iron for 4 weeks. Iron levels were increased with iron enriched feed analysed via LA‐ICP‐MS in the diaphragm muscle (*A*). Dietary modification did not change fibrosis (Van Gieson's) (*B*); superoxide (dihydroethidium; DHE) (*C*); or inflammation (CD68% nuclei) (*D*). Representative images of stains are shown in panel (*E*). Data presented as mean ± SEM. Data were analysed using Student's *t*‐test. *P ≤ 0.05. *N* = 8 in each group.

**Figure 5:**
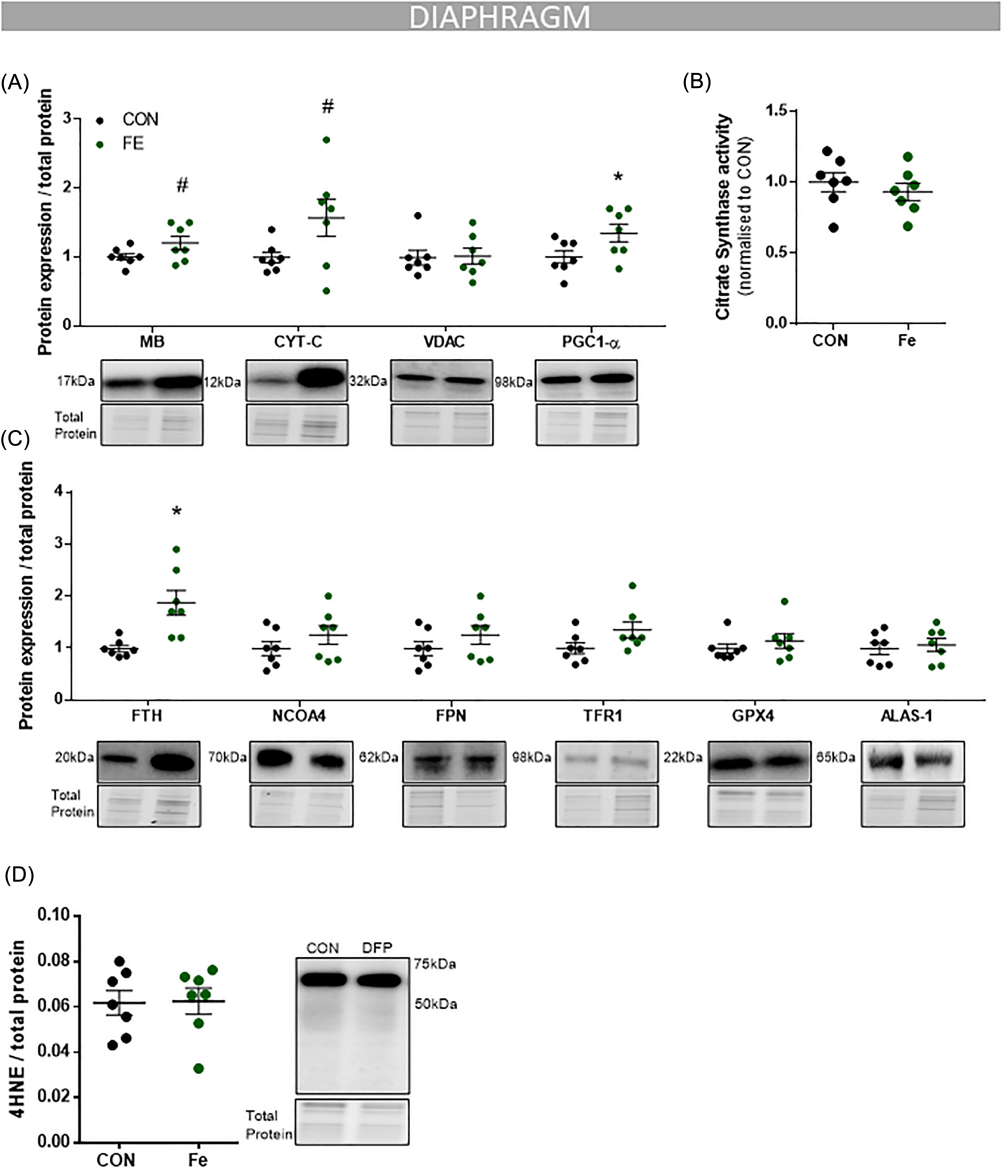
Fe treatment does not affect mitochondrial and haem containing proteins. *mdx* mice (4 weeks) were fed an iron enriched feed containing 1% added Fe as carbonyl iron for 4 weeks. Immunoblotting analysis in the diaphragm muscle showed that iron‐enriched feed increased two proteins that contain haem units; MB and CYT‐C and PGC‐1α, however, had no impact on mitochondrial marker; VDAC (*A*). Citrate synthase activity was also unchanged (*B*). There was a significant increase in ferritin (FTH), but no change in NCOA4, FPN, TFR1, GPX4 and ALAS‐1 (*C*), LC3BI, LC3BII, P62 (*D*), or 4HNE (*E*). Data presented as mean ± SEM. Data were analysed using Student's *t*‐test. *P ≤ 0.05. *N* = 8 in each group.

We also tested the hypothesis that increasing dietary iron would promote oxidative stress and pathology by increasing iron burden in muscle of the *mdx* mice. Dietary iron supplementation significantly increased total elemental iron in the diaphragm (+25%, *P* < 0.0205; *Figure* 4A). Despite elevated iron, there were no significant changes in growth (*Figure* S2A), body composition (*Figure* S2B), individual muscle mass (*Figure* S2C), grip strength (*Figure* S2D), blood glucose (*Figure* S2E), haematocrit (*Figure* S2F), superoxide levels (DHE: *Figure* 4C), fibrosis (Van Gieson's: *Figure* 4B), inflammation (CD68: *Figure* 4D), or pax7/Ki67**+** cells (*Figure* S4). The iron feeding induced an increase in the iron storage protein, ferritin (+87%, *P* < 0.05; *Figure* 5C). There were no changes in expression of proteins involved in ferritin breakdown, iron export and/or iron import (*Figure* 5C). The iron supplementation induced a trend towards increased haem‐containing proteins myoglobin (+20%, *P* < 0.1; *Figure* 5A) and cytochrome *c* (+57%, *P* < 0.1; *Figure* 5A), with no change in ALAS‐1 (*Figure* 5C). While the mitochondrial marker PGC‐1α was increased significantly (+35%, *P* < 0.05; *Figure* 5A), there were no significant differences in VDAC (*Figure* 5A) or citrate synthase enzyme activity (*Figure* 5B).

## Discussion

Iron‐generated reactive oxygen species might contribute to the oxidative stress that occurs in multiple models of muscle atrophy.^25^ Iron overload has been characterized in age‐related muscle wasting (sarcopenia),^6^ but whether iron accumulation also occurs in muscular dystrophy has received limited attention. DMD is the most severe and common of the muscular dystrophies and we investigated the role of iron homeostasis in the dystrophic pathology using two well‐characterized murine models of DMD. We found iron content was elevated in severely affected muscles, including the hind limb muscles of *dko* (*dystrophin/utrophin null*) mice and the diaphragm muscles of *mdx* mice. To further investigate the role of iron in DMD, we modulated iron via two different four‐week interventions (iron chelation vs. iron enriched diet). Treatment with the iron chelator DFP reduced oxidative stress and attenuated fibrotic deposition in dystrophic muscle and reduced mitochondrial oxidative activity and the abundance of haem‐containing proteins involved in oxidative metabolism. In contrast, increasing iron availability elevated the abundance of haem‐containing proteins but had no effect on oxidative stress or fibrosis, possibly due to a concomitant increase in iron storage protein, ferritin. These findings provide new insights into iron overload in DMD, and the involvement of iron in the dystrophic pathology.

Iron has a complex role in the regulation of cellular homeostasis. Free iron can drive the production of potentially harmful ROS, but haem‐iron is essential for oxygen transport and cellular respiration.^26^ As such, complex regulatory mechanisms control the amount and location of intracellular iron.^27^ Expression of genes involved in iron uptake, storage, and utilization, are controlled by iron availability which regulates the binding of iron‐responsive proteins (IRPs) to iron responsive elements (IREs), structural motifs within the untranslated regions of mRNAs (reviewed extensively previously^28^). In conditions of iron overload, an abundance of iron causes IRP1 to exist as an cytosolic aconitase and lose its affinity to the IRE, thus promoting translation of ferritin mRNA.^29^ This safety mechanism ensures that excess free iron can be stored in a ‘safe’ redox state with the ferritin multimer cage. Similarly, synthesis of the iron export protein ferroportin is induced by iron abundance.^30^ We found that iron overload in mouse models of DMD was associated with an increased abundance of ferritin and ferroportin. Thus, muscles of dystrophic mice appear to be compensating for iron overload by increasing iron storage and export, while redirecting free iron away from the cytoplasm by decreasing haem synthesis enzyme ALAS‐1 and haem‐containing proteins, myoglobin, and cytochrome *c*.

ALAS‐1 also contains an IRE and is usually upregulated with functional/free iron abundance,^28^ but impaired haem biosynthesis in the condition of iron overload has been observed previously. In zebrafish models with a disruption in the *Glrx5* gene encoding glutaredoxin 5, there is a constitutive activation of IRP1 for IRE‐binding and consequential down regulation of haem biosynthesis despite iron abundance.^31^ An alternative explanation lies with the inability to access stores of iron within ferritin. The ferritin multimer cage can contain 4500 atoms of iron and serves as temporary storage that can be released by selective autophagic degradation initiated by nuclear receptor coactivator 4 (NCOA4).^32^ NCOA4 binds to ferritin to allow trafficking to the lysosome for degradation via autophagy (ferritinophagy). Several studies have shown that autophagy is impaired in muscles of *dko/mdx* mice and in DMD patients,^33^, ^34^ evidenced by reduced expression of the autophagy marker MAP 1LC3B/LC3B (microtubule associated protein 1 light chain 3 beta) and increased accumulation of the protein SQSTM1/p62. It is also possible that our observation of iron overload is a consequence of impaired autophagy leading to impaired iron recycling and functional iron deficiency. Ferritin is usually degraded to release iron in times of deprivation,^35^ but DFP treatment did not reduce ferritin, NCOA4, LC3B or SQSTM1/p62, despite a reduction in iron and haem‐containing proteins. This raises the hypothesis that ferritin breakdown is impaired in dystrophic skeletal muscles.

To further elucidate impairments in iron handling in dystrophic models, we treated *mdx* mice with an iron chelator. In other models of iron overload such as neurodegeneration,^36^ macular degeneration,^37^ and beta thalassemia,^38^ iron chelation improves pathology, possibly by lowering the catalyst for ROS production. We also showed that reducing iron availability in the diaphragm of *mdx* mice reduced the generation of superoxide, which could contribute to oxidative stress. Evidence indicates that oxidative stress induces the expression of various cytokines and growth factors involved in fibrosis.^39^ Fibrosis is a hallmark of the dystrophic pathology, causing muscle dysfunction and forming a physical barrier for many potential therapies.^40^ We observed that the dampened superoxide in DFP treated mice was associated with reduced fibrosis, without apparent changes in mRNA expression in factors controlling fibrosis. Anti‐fibrotic therapies are of interest as co‐treatments with gene and cell therapies, but chelating iron to reduce ROS and fibrosis may be limited by the off‐target effects in haem containing proteins. Iron chelation with DFP decreased expression of mitochondrial markers including haem‐containing proteins such as cytochrome *c* and myoglobin, consistent with decreased mitochondrial activity. Interestingly, haem‐containing proteins that play essential roles in oxidative metabolism appeared to be sacrificed for iron chelation under these conditions, while ferritin was preserved. This is of interest also because iron deficiency has been associated with reduced myoglobin and cytochrome *c* resulting in impaired skeletal muscle oxidative capacity.^41^

To our knowledge, no study has examined the impact of increasing intramuscular iron by a dietary intervention in either healthy or dystrophic mice. Iron supplementation of weakened anaemic patients improved muscle strength and resistance to fatigue.^42^ Similarly, in 1965, Dallman investigated the impact of iron deficiency and subsequent iron repletion on a range of tissues in male rats.^43^ Iron deficiency caused anaemia, decreased cytochrome *c* in multiple tissues, and lowered myoglobin in muscle. Interestingly, with subsequent iron supplementation, intestinal mucosa derived cytochrome c returned to control levels within 2 days despite the rats remaining anaemic, but the time required for the rescue of myoglobin and cytochrome *c* in skeletal muscle was five times longer, indicating the need for assessments of iron homeostasis to be performed in a tissue‐specific manner.^43^ In this study, we also showed that iron feeding in non‐anaemic dystrophic mice increased cytochrome *c* and myoglobin, potentially rescuing a functional iron deficiency.

The iron supplemented tissues also seem to have responded through conventional IRE/IRP mechanisms leading to the increase in ferritin to safely store iron as described previously.^28^ This might have been a protective mechanism by lowering superoxide generation and, consequently, fibrosis. It is interesting to note that ferritin levels only responded by elevating to iron overload, and not decreasing upon iron chelation. This may have been due to an inadequate dose of DFP, or this response could reflect disease‐specific changes where the muscle can increase iron storage without being able to access and recycle ferritin‐bound iron, potentially rendering iron supplementation futile as a therapy for dystrophic pathology. Future therapies could focus on increasing the functional iron pool for incorporation into haem proteins to maintain or improve oxidative metabolism, potentially by restoring abnormal autophagy, while mitigating the harmful downstream complications of iron overload via antioxidants that inhibit lipid peroxidation and ROS.

## Conclusion

To our knowledge, we present the first description of iron overload and altered iron handling in two murine models of DMD. Dystrophic skeletal muscles showed increased total elemental iron and ferritin. Iron chelation with deferiprone at one dose ameliorated fibrosis but suppressed mitochondrial function. Conversely, iron feeding elevated iron and haem proteins, but had no impact on fibrosis. Thus, iron status impacts on the pathology of DMD and warrants further elaboration.

## Conflict of interest

Prof. Bush is a shareholder in Alterity Ltd, Cogstate Ltd, Brighton Biotech LLC, Grunbiotics Pty Ltd, Eucalyptus Pty Ltd, and Mesoblast Ltd. He is a paid consultant for, and has a profit share interest in, Collaborative Medicinal Development Pty Ltd.

## Supporting information


**Figure S1.** *mdx* mice (4 weeks) were given access to drinking water with or without deferiprone (DFP: 150 mg/kg/day; *n* = 10) for 4 weeks. There was no change in growth during the treatment period (A), body composition at the end of the 4 week intervention (B), final individual tissue masses (TA, EDL, QUAD, SOL, PLAN, GAST, FAT; or HEART) (C), maximum grip strength (g/kg) normalized to body mass (D) or blood glucose in response to a glucose tolerance test (area under the curve, AUC, E). Data presented as mean ± SEM. Data were analysed using Student's t‐test. *P ≤ 0.05 *n* = 10 in each group.
**Table S1.** *mdx* mice (4 weeks) were given access to drinking water with or without deferiprone (DFP: 150 mg/kg/day) for 4 weeks. Diaphragm muscles used for qPCR analysis of inflammatory mRNA showed a significant reduction in ***TNF*‐α** and increase in *F480* and a trend for decreased *Hmox*‐1. There was no change in other inflammatory mRNA including *Ccl2*, *Socs3, Il6* and *Cd80*. There was also no change in mRNA associated with fibrosis including *Col1a1*, *Col2a1*, *Col3a1*, *Mmp2*, *Mmp9*, *Tgfb*1, *Tgfb*2, *Tgfb*3, *Vegf*, *Timp1* and *Timp3*. Data presented as mean ± SEM. Data were analysed using Student's t‐test. *P ≤ 0.05. **#*P* ≤ 0.1 *n* = 10.**
**Figure S2.** *mdx* mice (4 weeks) were given access to drinking water with or without deferiprone (DFP: 150 mg/kg/day; n = 10) for 4 weeks. There was no change in Pax7/mm^2^ (A) or Ki67+/mm^2^ (B). Representative images of the diaphragm shown in (C). Data presented as mean ± SEM. Data were analysed using Student's t‐test. *P ≤ 0.05 *n* = 6 in each group.
**Figure S3.** *mdx* mice (4 weeks) were fed an iron enriched feed containing 1% added Fe as carbonyl iron for 4 weeks. There was no change in growth during the treatment period (A), body composition at the end of the 4‐week intervention (B), endpoint blood haematocrit (C), final individual tissue masses (TA, EDL, QUAD, SOL, PLAN, GAST, FAT; or HEART) (D), maximum grip strength (g/kg) normalized to body mass (E) or resting blood glucose (F). Data presented as mean ± SEM. Data were analysed using Student's t‐test. *P ≤ 0.05. *n* = 10 in each group.
**Figure S4.** Fe treatment does not affect Pax7/mm^2^ (A) or Ki67+/mm^2^ (B). Representative images of the diaphragm shown in (C). Data presented as mean ± SEM. Data were analysed using Student's t‐test. *P ≤ 0.05 *n* = 6 in each group.
