# Supplementary material for: Iron overload and impaired iron handling contribute to the dystrophic pathology in models of Duchenne muscular dystrophy
Source: J Cachexia Sarcopenia Muscle. 2022 Mar 6;13(3):1541–53. doi: 10.1002/jcsm.12950 (PMC9178167; doi:10.1002/jcsm.12950)

Supplementary Figure 1:

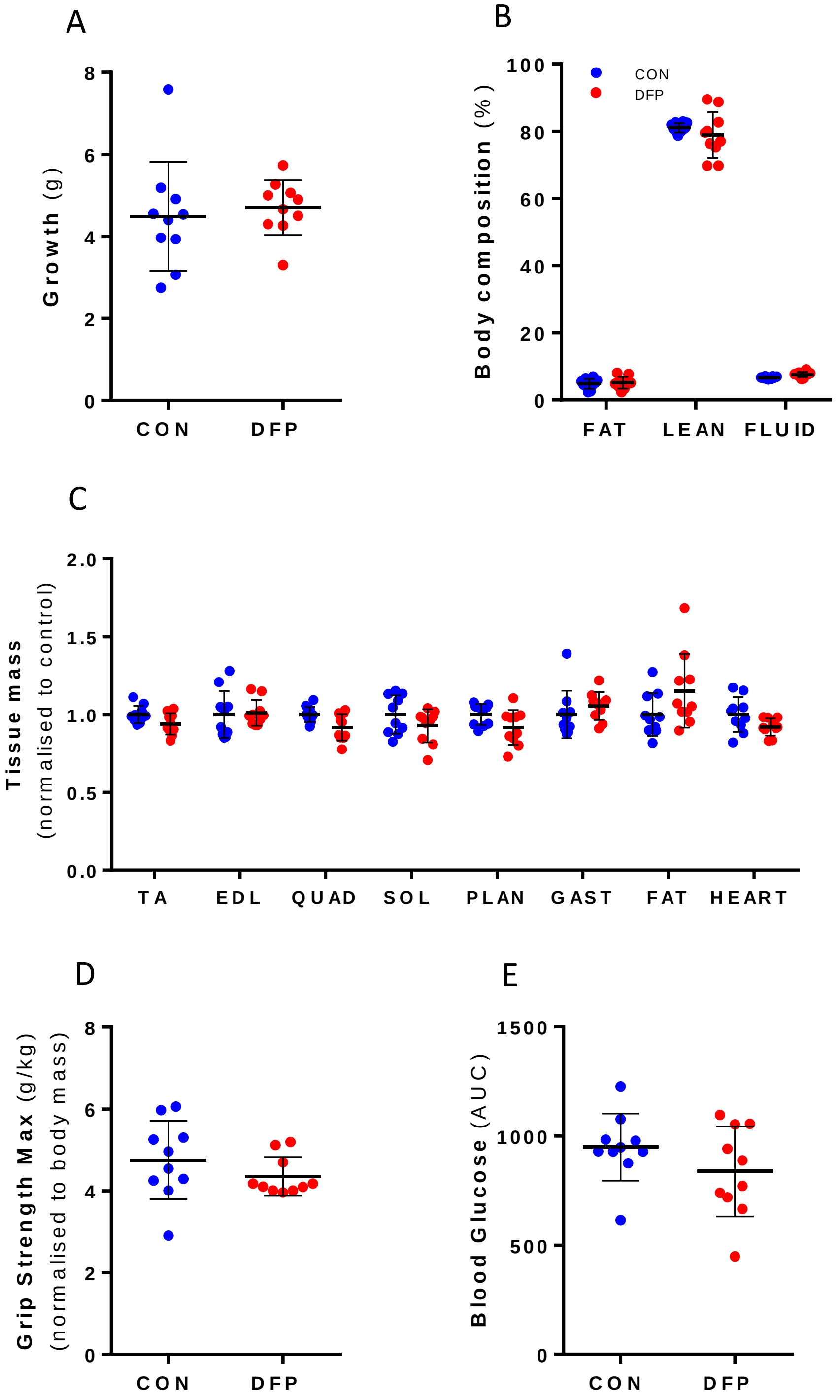

Supplementary Figure 2:

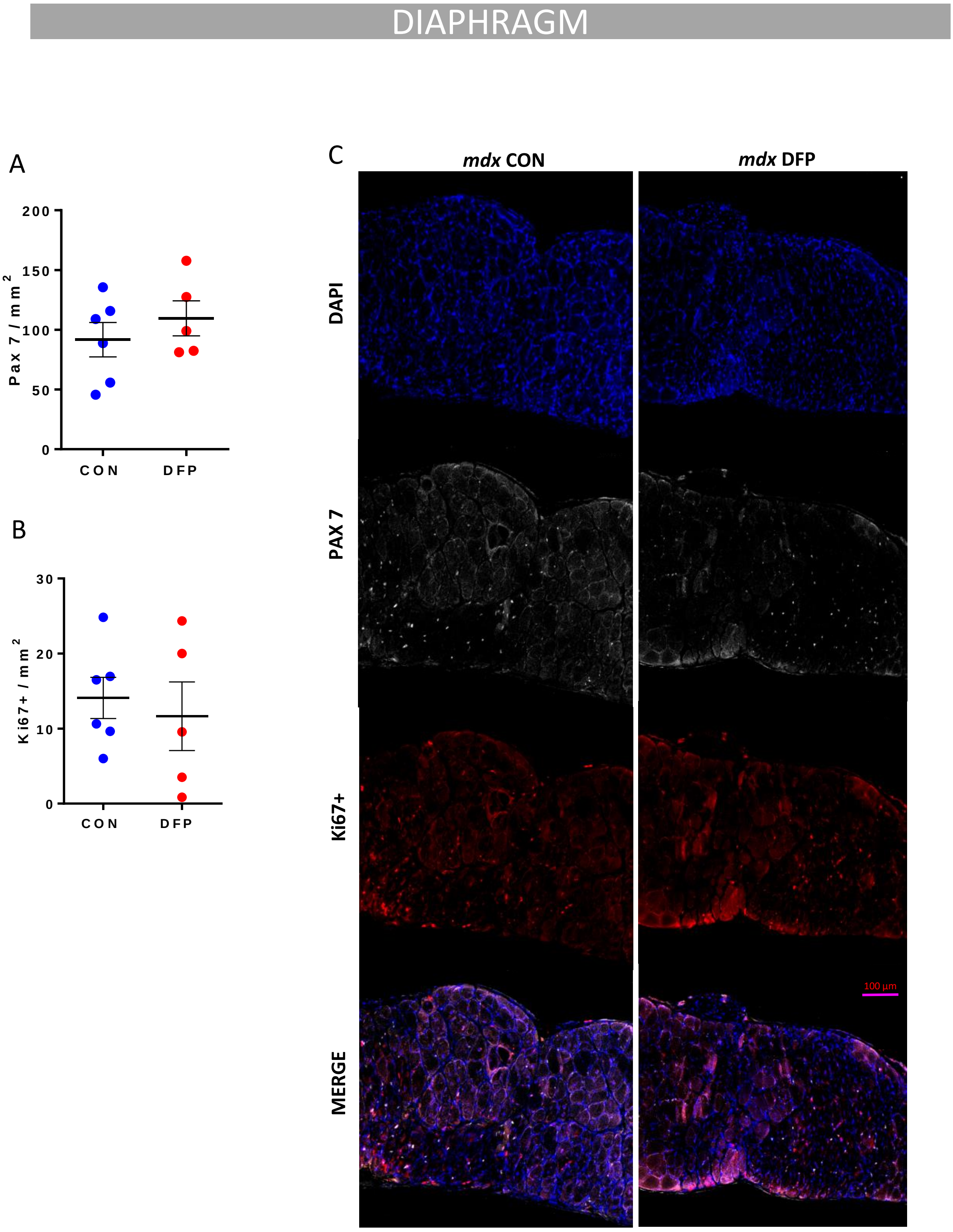

Supplementary Table 1: PCRs.

Gene	*MDX* DFP (Fold change)		
	Mean	±SEM	*P value*
Inflammation			
Ccl2	0.76	0.18	P=0.196
TNFa	0.65	0.14	*P=0.024
Socs3	1.01	0.30	P=0.966
Il6	1.79	0.44	P=0.087
CD80	1.71	0.34	P=0.052
F480	2.84	0.75	*P=0.024
Hmox-1	0.71	0.63	#P=0.06
Fibrosis			
Col1a1	1.04	0.18	P=0.825
Col2a1	1.02	0.64	P=0.864
Col3a1	0.76	0.18	P=0.135
Mmp2	0.97	0.21	P=0.810
Mmp9	0.70	0.42	P=0.476
Tgfb1	0.97	0.15	P=0.858
Tgfb2	0.99	0.07	P=0.840
Tgfb3	0.97	0.15	P=0.858
Vegf	0.78	0.13	P=0.096
Timp1	0.92	0.14	P=0.576
Timp3	0.88	0.14	P=0.393

Supplementary Figure 3:

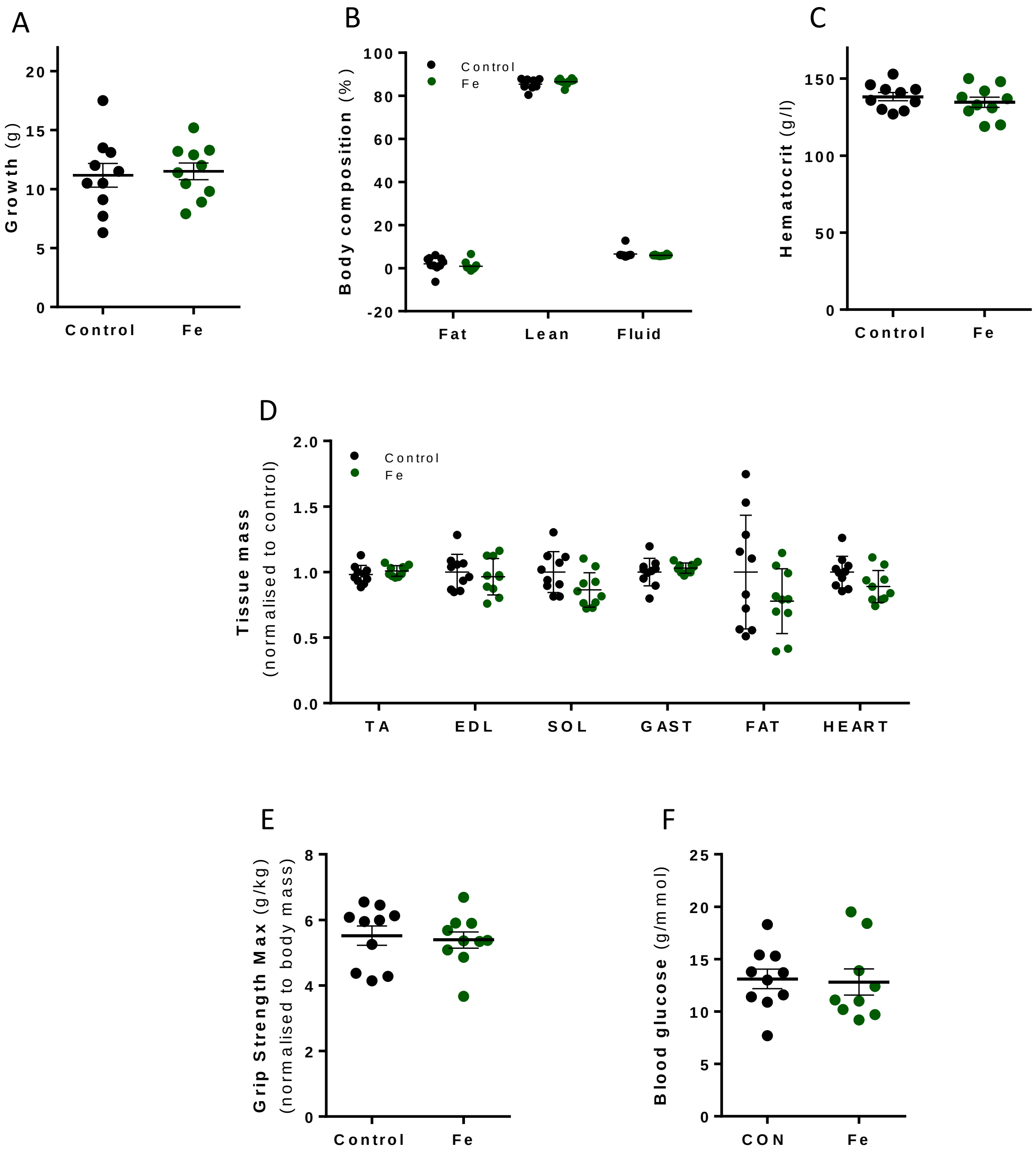

Supplementary Figure 4:

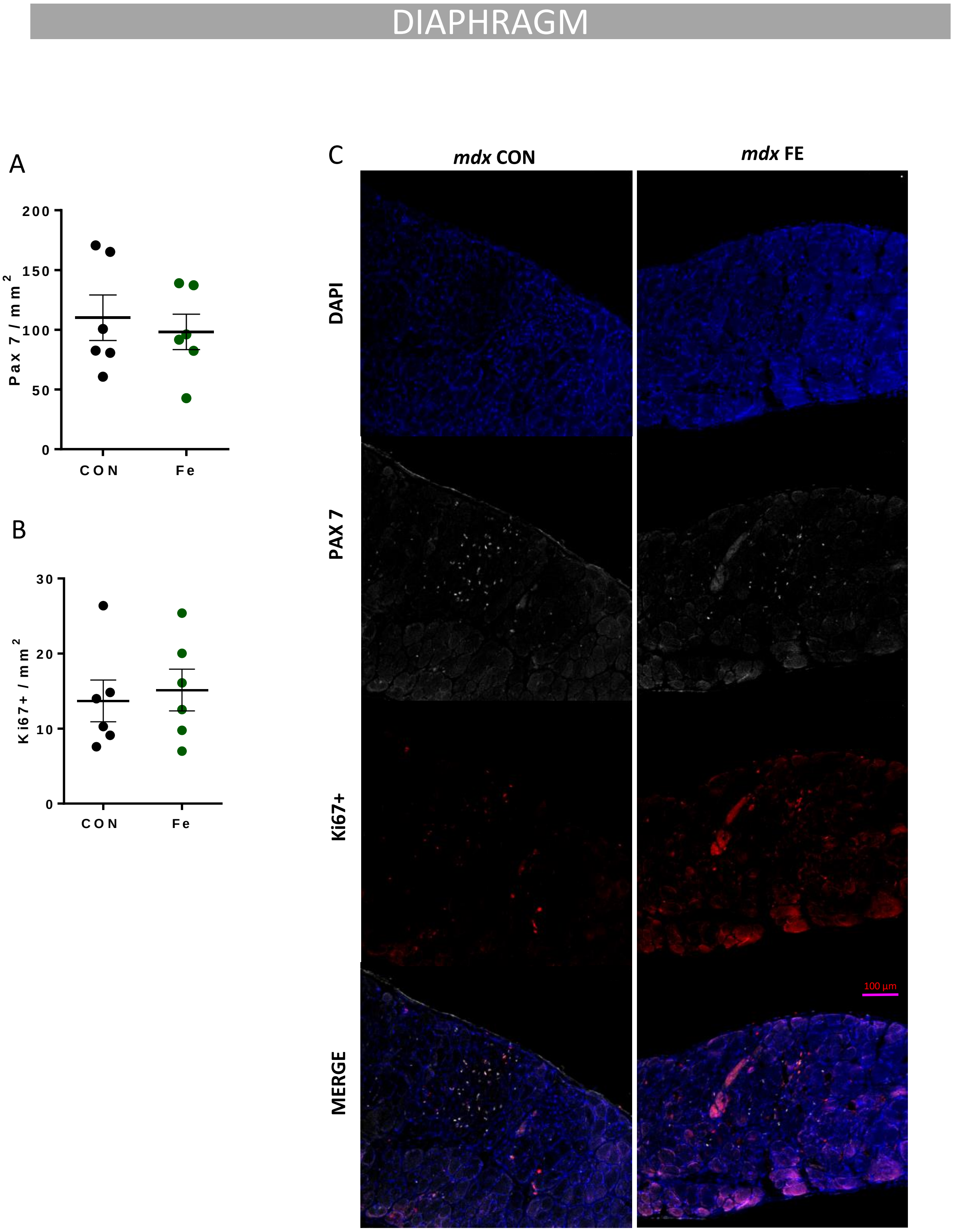

Supplement: Supplementary file 1 — Figure S1. mdx mice (4 weeks) were given access to drinking water with or without deferiprone (DFP: 150 mg/kg/day; n = 10) for 4 weeks. There was no change in growth during the treatment period (A), body composition at the end of the 4 week intervention (B), final individual tissue masses (TA, EDL, QUAD, SOL, PLAN, GAST, FAT; or HEART) (C), maximum grip strength (g/kg) normalized to body mass (D) or blood glucose in response to a glucose tolerance test (area under the curve, AUC, E). Data presented as mean ± SEM. Data were analysed using Student's t‐test. *P ≤ 0.05 n = 10 in each group. Table S1. mdx mice (4 weeks) were given access to drinking water with or without deferiprone (DFP: 150 mg/kg/day) for 4 weeks. Diaphragm muscles used for qPCR analysis of inflammatory mRNA showed a significant reduction in TNF‐α and increase in F480 and a trend for decreased Hmox‐1. There was no change in other inflammatory mRNA including Ccl2, Socs3, Il6 and Cd80. There was also no change in mRNA associated with fibrosis including Col1a1, Col2a1, Col3a1, Mmp2, Mmp9, Tgfb1, Tgfb2, Tgfb3, Vegf, Timp1 and Timp3. Data presented as mean ± SEM. Data were analysed using Student's t‐test. *P ≤ 0.05. #P ≤ 0.1 n = 10. Figure S2. mdx mice (4 weeks) were given access to drinking water with or without deferiprone (DFP: 150 mg/kg/day; n = 10) for 4 weeks. There was no change in Pax7/mm2 (A) or Ki67+/mm2 (B). Representative images of the diaphragm shown in (C). Data presented as mean ± SEM. Data were analysed using Student's t‐test. *P ≤ 0.05 n = 6 in each group. Figure S3. mdx mice (4 weeks) were fed an iron enriched feed containing 1% added Fe as carbonyl iron for 4 weeks. There was no change in growth during the treatment period (A), body composition at the end of the 4‐week intervention (B), endpoint blood haematocrit (C), final individual tissue masses (TA, EDL, QUAD, SOL, PLAN, GAST, FAT; or HEART) (D), maximum grip strength (g/kg) normalized to body mass (E) or resting blood glucose (F). Da [file JCSM-13-1541-s001.pdf]
